# Comparative Analysis of the LOBO Device and Coils for Proximal Splenic Artery Embolization: A Multi-Institutional Retrospective Study

**DOI:** 10.1007/s00270-026-04460-0

**Published:** 2026-05-12

**Authors:** Roshan Valentine, Massoud Allahyari, Johannes L. du Pisane, Chaitanya Ahuja, Alexander Villalobos, Harrison Bieber, Nicholas Thomas, Ali Afrasiabi, Bahareh Gholami, Sandra Gad, Gloria Salazar, David Mauro, Nima Kokabi

**Affiliations:** 1 Division of Vascular & Interventional Radiology, Department of Radiology, University of North Carolina at Chapel Hill, 101 Manning Drive, Chapel Hill, NC 27514 USA; 2 Division of Interventional Radiology, Department of Radiology, Louisiana State University Health Sciences Center-Shreveport, Shreveport, LA USA; 3 St. George’s University, West Indes, Grenada

**Keywords:** Splenic trauma, Proximal splenic artery embolization, Plug, Coil, LOBO

## Abstract

**Purpose:**

To evaluate the technical and clinical efficacy of the low-profile braided occluder (LOBO) device for proximal splenic artery embolization (SAE) and compare its performance with conventional coil embolization.

**Materials and Methods:**

This retrospective multi-institutional study included 44 consecutive patients who underwent proximal SAE between June 2023 and October 2025. Demographic data, etiology, embolic device type, fluoroscopy time, radiation dose, and outcomes were recorded. Technical success was defined as complete angiographic occlusion using the index device. Clinical success was defined as the absence of rebleeding or reintervention within 30 days.

**Results:**

Of the 44 patients (mean age, 43.2 years; 39% females), 22 were treated using the LOBO device and 22 with coils. Trauma was the most common etiology (*n* = 37, 84.1%). There were no clinical or demographic characteristic differences between the LOBO and coil groups. Proximal SAE was achieved with a single LOBO device in all patients. The mean number of coils used was 4.6 per case (median 3.5), *p* < 0.001. Median fluoroscopy time was significantly reduced with LOBO compared with coils (11.65 vs 18.9 min; *p* = 0.01). Radiation dose was significantly lower as well in the LOBO group compared with the coil group (128.4 vs 198.5 mGy; *p* = 0.04). Clinical success was not significantly different between the groups, with the LOBO group achieving 100% and the coil group 90.9% (*p* > 0.05). Splenic infarction was significantly higher with coils than with LOBO (33.3% vs 22.7%; *p* = 0.046). No major adverse events occurred, and minor events were self-limited. One death in each group was attributable to polytrauma and unrelated to the procedure.

**Conclusion:**

The LOBO device provides safe, effective proximal SAE with significantly reduced fluoroscopy time and radiation dose compared with coils. Larger prospective studies are warranted.

**Level of Evidence:**

Level 4, study (retrospective, standard quality).

**Graphical Abstract:**

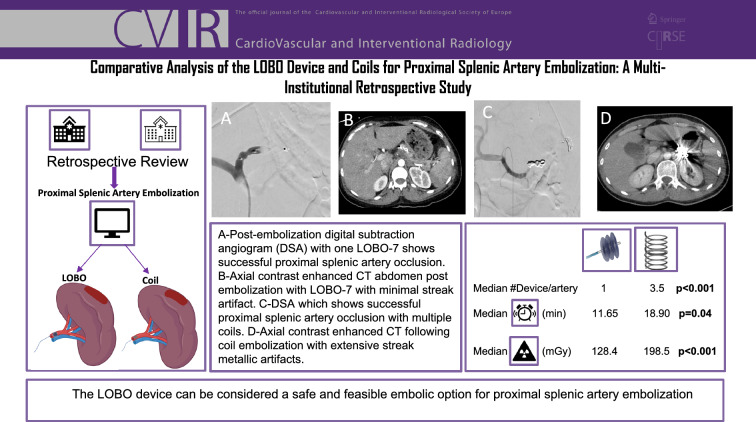

## Introduction

Splenic injury is the most common solid organ injury associated with blunt abdominal trauma [1]. In the late 1970s, there was a shift from operative to nonoperative management of splenic injury prompted by the observed increased infection rates after splenectomy [2]. Splenic artery embolization (SAE), first performed by Sclafani in 1981, utilized steel wool coils, gelfoam, and a vasopressin infusion to achieve embolization; the technique has since evolved with the introduction of multiple embolic options on the market [3]. These include pushable and detachable coils, vascular plugs, and various liquid embolic agents. SAE can be performed either proximally or distally, depending on the indication for embolization. Proximal embolization is often performed in traumatic splenic hemorrhage in the setting of multifocal injuries or when angiography shows no active arterial extravasation but CT demonstrates visceral injury [4]. Distal SAE is often performed for focal splenic bleed with active arterial extravasation, in portal hypertension to decrease portal pressure, or in hypersplenism to reduce splenic volume.

An effective embolic device ensures durable vessel occlusion while minimizing procedure time and radiation exposure. The low-profile braided occluder (LOBO) (Okami Medical, Aliso Viejo, California, USA) is a new addition to the sphere of embolic devices and was approved by the US Food and Drug Administration (FDA) in 2020 for embolization in peripheral arteries. Early reports have described promising technical results using LOBO in patients with pulmonary arteriovenous malformations and in a recent trauma-related case series of three patients who underwent splenic artery embolization (SAE) [5, 6]. However, given the very small sample sizes and the device's relative infancy, these data provide only low-level evidence and preclude definitive conclusions regarding safety, durability, or comparative efficacy.

Among available embolic options, coils remain among the most commonly used and most extensively studied embolic for proximal SAE and therefore serve as the most clinically relevant device for comparison.

Accordingly, this multi-institutional retrospective study aims to evaluate the technical and clinical efficacy of LOBO in SAE and to compare procedural efficiency and outcomes with those of conventional coil embolization [7].

## Materials and Methods

This retrospective multi-institutional (2 institutions) study was conducted after obtaining Institutional Review Board approval at both participating centers in accordance with the Health Insurance Portability and Accountability Act of 1996 (HIPAA) and institutional policies governing the protection of patient privacy.

The respective hospital databases were queried for all patients of SAE between June 2023 and October 2025. Indications for splenic artery embolization included American Association of the Surgery for Trauma (AAST) Grade ≥ 2, CT evidence of active extravasation or pseudoaneurysm, or large hemoperitoneum. Patients with uncorrectable coagulopathy, distal splenic artery embolization only, and pregnant patients were excluded. Distal SAE alone, in particular, was excluded to preserve procedural and clinical comparability and reduce heterogeneity. Distal and proximal approaches differ in complication rates, with proximal SAE generally associated with fewer, typically minor complications [8]. Data collected included patient demographics (age, sex), clinical indication, number and type of embolic devices used, adjunctive embolic agents/devices, fluoroscopy time, and total radiation dose.

**Fig. 1:**
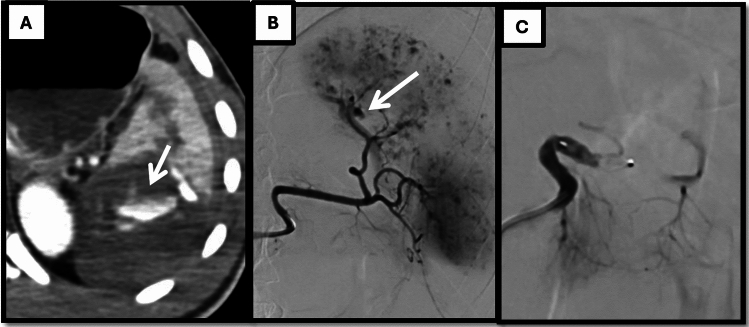
**A** Axial contrast-enhanced CT (CECT) of the abdomen in a 22-year-old male status post motor vehicle accident shows splenic laceration with active contrast extravasation (arrow). **B** Selective splenic arteriogram demonstrates a tortuous splenic artery with multiple pseudoaneurysms within the splenic parenchyma (arrow) **C** Post-embolization digital subtraction angiogram (DSA) with LOBO-7 shows successful proximal splenic artery occlusion with delayed opacification of the spleen via collateral vessels

**Fig. 2:**
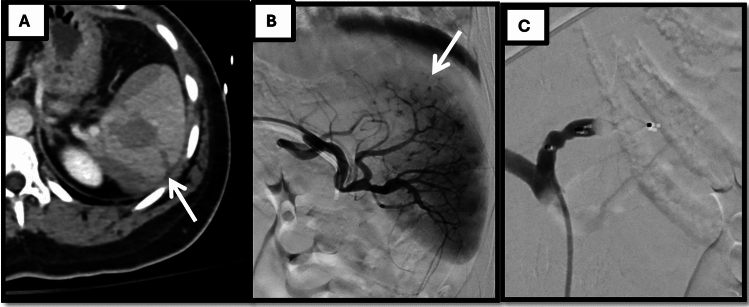
**A** Axial contrast-enhanced CT (CECT) of the abdomen in a 36-year-old female status post motor vehicle accident shows splenic laceration and perisplenic hematoma (arrow). **B** Selective splenic arteriogram demonstrates a tortuous splenic artery with multiple pseudoaneurysms within the splenic parenchyma (arrow) **C** Post-embolization digital subtraction angiogram (DSA) with LOBO-7 shows successful proximal splenic artery occlusion

Conscious sedation or general anesthesia was administered based on hemodynamic status. Pre-procedural CT was performed for all patients except for those who were hemodynamically unstable, and point-of-care ultrasound confirmed hemoperitoneum. Access was obtained via the right or left common femoral artery or the left radial artery under sterile technique, using a 5–6 Fr sheath (Terumo inc, Japan). After catheterization of the parent artery utilizing a 5 Fr to 5.5 Fr diagnostic catheter and guidewire, an angiogram was performed to localize the site of bleeding. LOBO size selection was based on the target vessel diameter, as determined intra-procedurally by angiography, per the manufacturer’s guidelines. The smallest device in the LOBO family, the LOBO-3, can be deployed through 2.8Fr microcatheters and embolized arteries as small as 1.5–3 mm. The LOBO-5 can be deployed through a 2.9 Fr microcatheter for occluding vessels with diameters of 3–5 mm. The LOBO-7 device requires a 5–5.5 Fr guiding catheter and is suitable for vessels measuring 5–7 mm in diameter, and the LOBO-9 device is useful for arteries with a diameter measuring 7–9 mm and can be deployed using a 5.5–6 Fr guiding catheter (Figs. 1 and 2). No additional oversizing was performed for LOBO, whereas the initial anchoring coil was typically oversized, approximately 30–50% relative to the target vessel diameter. According to the surgeon’s preference, some patients with post-traumatic splenic injury underwent pre-emptive embolization prior to splenectomy. Twelve interventional radiologists, with 3–20 years of experience, performed procedures using comparable fluoroscopy systems at both institutions. Device choice was determined by the treating interventional radiologist based on anatomic and technical factors, including vessel tortuosity, operator preference, and device availability.

The primary endpoints were technical success, defined as complete angiographic occlusion of the splenic artery using the index device, and clinical success, defined as cessation of hemorrhage without reintervention or evidence of bleeding within 30 days in those undergoing proximal SAE for hemorrhage. Other comparative endpoints included splenic infarction in patients with available follow-up imaging, fluoroscopy time, and radiation dose. Splenic infarction was assessed on follow-up CT when available; routine surveillance CT was not uniformly performed in all patients. Fluoroscopy time and radiation dose were recorded from procedural data. Complications were defined according to the Cardiovascular and Interventional Radiological Society of Europe (CIRSE) classification system [9]. All statistical analyses were performed using SPSS Statistics (IBM Corp., Armonk, NY, USA). Categorical variables, including sex, bleeding etiology, and type of embolic device used, were expressed as frequencies and percentages. Continuous variables were expressed as mean ± standard deviation (SD) or median (interquartile range, IQR) based on data distribution. Comparisons between groups were performed using the Mann–Whitney U test for non-parametric data. P-value less than 0.05 was considered significant. A post hoc sample-size justification was conducted for the continuous endpoint representing embolic device utilization per case. Assuming a mean of 1 device in the LOBO group versus 2 devices in the coil group, a common standard deviation of 1, a two-sided α of 0.05, and 90% power, the required sample size was 21 patients per group. Our study included 22 patients per cohort, for a total sample size of 44 patients, thereby meeting the prespecified threshold and providing adequate power for this comparison.

## Results

### Patient Cohort and Indications

Forty-four patients underwent SAE (mean age, 43.22 years; 27 males, 17 females). Twenty-two were treated using LOBO, and twenty-two using coils. The most common etiology was trauma (*n* = 37, 84.1.5%), followed by iatrogenic causes (*n* = 3, 6.8%), pancreatitis (*n* = 3, 6.8%), and spontaneous splenic rupture (*n* = 1, 2.3%).

The LOBO and coil groups were similar in age (41.77 ± 24.93 vs 41.47 ± 18.4 years; *p* value = 0.65) and sex distribution (14:8 vs 13:9 *p* value = 1). Hemodynamic status on presentation was comparable, with hemodynamically stable patients in 63.6% (14/22) of LOBO patients and 68.2% (15/22) of coil patients. Mean splenic artery diameter was 4.25 ± 1.01 mm in the LOBO group and 4.90 ± 2.22 mm in the coil group (Table 1). Injury severity was comparable between groups, with no statistically significant difference in AAST grade distribution (*p* = 0.98) (Table 1). Two patients in each cohort underwent tandem embolization when angiography demonstrated additional splenic injury.

**Table 1:** Baseline cohort characteristics

Variable	Coil (*n* = 22)	LOBO (*n* = 22)	*P*-value
Age (years)	42.3 ± SD	43.6 ± SD	0.79
Sex (Male/Female)	14 / 8	13 / 9	1.00
Splenic artery diameter (mm)	Median 4.1 (IQR 3.4–4.7)	Median 5.0 (IQR 4.0–5.8)	0.13
AAST Grade II	2	2	
AAST Grade III	7	7	
AAST Grade IV	10	9	
AAST Grade V	3	4	0.98

*AAST* American Association for the Surgery of Trauma

### Devices Used

Proximal splenic artery embolization was successfully embolized with 1 device in all patients in the LOBO cohort. Specifically, 9 patients received LOBO-5, 12 received LOBO-7, and one received LOBO-9.

**Fig. 3:**
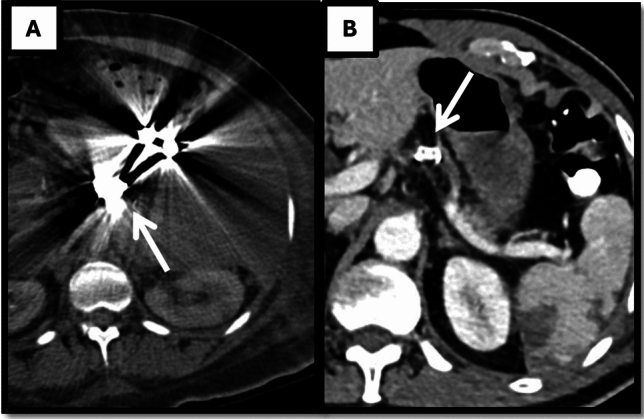
**A** Axial unenhanced CT abdomen of a 49-year-old male following coil embolization of an idiopathic splenic artery aneurysm demonstrating extensive streak metallic artifacts from the coils (arrows) that obscure the diagnostic quality of the image. **B** Axial CECT abdomen of a 66-year-old male following splenic artery embolization with a LOBO device, demonstrating the device in situ with no significant metallic artifact and clear delineation of the surrounding parenchyma (arrows)

In the coil group, a total of 111 coils were used with a mean of 4.6 coils/patient (median, 3.5; range, 1–19; SD, 4.27) (Figure 3). Specifically, 69 Nester coils (Cook Incorporated, Bloomington, IN, USA), 24 Embold fibered coils (Boston Scientific, USA), 11 Embold soft coils, 2 Embold packing coils, 2 Tornado coils (Cook Incorporated, Bloomington, IN, USA), 2 Azur coils (Terumo Medical Corp., Somerset, New Jersey), and 1 MReye coils (Cook Incorporated, Bloomington, IN, USA).

### Procedural Characteristics

Median fluoroscopy time was shorter with the LOBO device compared to coils (median [IQR], 11.65 [7.95–16.4] min vs 18.9 [13.08–22.7] min; *p* = 0.01). Radiation dose was significantly lower in the LOBO group compared with the coil group, with median values of 128.4 (85.1–190.6) mGy versus 198.5 (145.2–265.8) mGy (*p* = 0.04) (Table 2). Additionally, device utilization was significantly lower in the LOBO group with an average of 1 device compared with an average of 4.6 coils in the coil group (median 1 LOBO vs. 3.5 coils; *p* = 0.0003).

**Table 2:** Procedural differences between LOBO and coils cohort

Procedure characteristics	Mean ± SD	Median(IQR)	Range	*p*- value(outlier excluded)
Fluoroscopy time (minutes)				
LOBO	13.18 ± 6.69	11.65 (7.95–16.45)	5.5 – 27.2	0.01*^+^
Coils	19.19 ± 8.34	18.9 (13.1–22.7)	6.8 – 44.1	
Radiation dose (mGy)				
LOBO	237.97 ± 271.56	128.4 (85.1–190.6)	67.8 – 1115.27	0.04*^+^
Coil	257.05 ± 183.10	198.5 (145.2–265.8)	51.1 – 552.0	

*p-values derived from Mann–Whitney U test

^+^Significant after outlier exclusion

Outlier removed table

Technical and clinical success was 100% for LOBO, with no reintervention or rebleeding within 30 days of embolization. Specifically, all 22 patients undergoing SAE for bleeding with LOBO remained free of spleen-related rebleeding at 30 days. The technical success in the coil group was also 100%. However, 2 out of 22 patients with bleeding undergoing SAE with coils had rebleed, which required re-embolization, resulting in a clinical success rate of 90.9%.

Of note, 3 patients underwent splenectomy due to trauma surgeon’s preference rather than for concerns of rebleeding (one in the LOBO group and two in the coil group) and were excluded from the clinical success analysis. Two patients in the coil group experienced rebleeding, both of which were successfully managed with repeat embolization. In the LOBO cohort, two patients underwent tandem embolization: one received a proximal LOBO with distal LAVA liquid embolic, and the other received a proximal LOBO with a distal Nester coil to ensure complete occlusion of distal arterial feeders before reducing splenic parenchymal pressure through proximal embolization. Similarly, two patients in the coil cohort underwent tandem embolization following splenic trauma. These adjunct embolic agents were used for embolization of distal splenic artery branches.

### Adverse Events

Minor complication (grade 1b) consisted of pain (*n* = 2) and nausea (*n* = 1), with pain occurring in the coil group and nausea in the LOBO group. All events were managed conservatively. There was one death in each group, both attributable to polytrauma and unrelated to the procedure. The mean duration of imaging follow-up was 59.5 ± 78.8 days. The proportion of patients without documented follow-up imaging was similar between cohorts (38.1% vs 40.9%; *p* = 1.0). Among patients with available follow-up imaging, splenic infarction (grade 3b) occurred more frequently in the coil cohort than in the LOBO cohort (33.3% vs 22.7%; *p* = 0.046). Splenic abscesses (grade 3b) occurred at the same rate in both groups (4.5% vs 4.5%; *p* = 1.0). Splenectomy within 30 days was performed in two coil-treated patients and one LOBO-treated patient (*p* = 1.0). Three patients underwent splenectomy post-embolization at the surgical team’s discretion despite hemostasis. Additionally, there was no device-related adverse event, including migration of the detached LOBO device in the proximal splenic artery.

## Discussion

This multi-institutional study demonstrates that the LOBO device is potentially safe and effective option for proximal splenic artery embolization, achieving 100% technical and clinical success, while significantly reduced number of devices used, fluoroscopy time and radiation dose compared with coils.

The selection of embolic agents remains crucial for achieving successful outcomes in the management of splenic injuries. This decision is based on various patient factors, including vessel anatomy, injury type, and embolization site. Coils, temporary embolic agents, vascular plugs, and liquid embolic agents each have their strengths and limitations with each use case in SAE [10–14]. Coils are commonly utilized in SAE. Technical success of coils has been shown to range from 88 to 100%, while the clinical failure rates vary between 12.1 and 27% [15]. They can be deployed through large and small French size catheters based on the vessel size and can be either pushable or detachable. Most often, multiple coils are required to occlude larger high-flow arteries like the splenic artery [7]. A meta-analysis by Johnson et al. showed that an average of 3.54 coils was used per SAE, which is in concordance with our study, where a mean of 4.6 coils was utilized per SAE [7]. Coils along with the temporary embolic agent such as gelfoam have shown varying primary clinical success ranging between 73 and 92.9% across various studies [15]. Coil migration is one of the complications associated with coil embolization, especially in a high-flow system such as the splenic artery; with reported coil migration rate of 31.8% [16]. In the present study, LOBO achieved complete occlusion with a single device and was associated with reductions in both fluoroscopy time and radiation dose, including a mean fluoroscopy time difference of 7.32 min (*p* = 0.012) and a median radiation dose difference of 70.1 mGy, supporting its procedural efficiency compared with coil embolization (Fig. 1). It is worth noting that two patients in both groups underwent tandem embolization of the distal splenic artery based on angiographic findings; therefore, it is unlikely to have skewed results for a particular cohort. Finally, radiation dose is influenced by multiple factors related to procedural complexity and cannot be attributed solely to device choice.

Vascular plugs are an effective alternative to coils for proximal SAE. A meta-analysis comparing the Amplatzer vascular plug (AVPs) (St. Jude Medical, Inc., St. Paul, MN, USA), with coils showed on average, in the AVP group, 4.43 devices (additional AVPs or coils) were required per procedure compared to 9.97 coils, with a shorter procedure time (55.6 min vs 45.82 min). The reported technical success rate for AVP in proximal SAE ranges from 87 to 100% and clinical success (splenic salvage) from 98 to 100% [17]. However, multiple embolic devices AVPs are often necessary for complete occlusion, and deployment can be challenging in tortuous vessels due to the device’s relative stiffness and stainless-steel core [6, 16, 18]. In comparison, all the LOBO procedures in this study only required a single device without the need for any additional embolic. Similar to devices such as the AVPs, the LOBO device does not produce significant imaging artifacts as compared to coils, especially on CT, which is another advantage, especially when analyzing follow-up imaging [19].

Micro vascular plugs (MVPs) are another routinely utilized device in arterial embolization. Their lower recanalization rates and ease of deployment compared with other metallic embolic devices, such as coils and AVPs, have made them a suitable choice for embolization in certain situations. In a multicenter retrospective study by Giurazza et al. evaluating the efficacy of MVP in the management of arterial hemorrhage, an overall technical success rate of 75% and a primary clinical success rate of 96.1% were reported [20]. Furthermore, a study by Mailli et al. showed the need for reinforcement with additional devices in 20 of 30 patients treated for arterial hemorrhage treated with MVP [21]. Therefore, both aforementioned studies demonstrate that a significant proportion of patients require additional embolic devices to achieve complete occlusion when using MVP for the management of arterial hemorrhage.

Splenic infarction and abscess formation remain key concerns following SAE. Few studies have criticized distal SAE for splenic abscess formation and splenic infarcts [15, 22]. A retrospective study of 72 patients showed that combined proximal and distal embolization was associated with a higher rate of splenic abscess and splenic infarction than either proximal or distal embolization [23]. A retrospective study demonstrated that proximal embolization was associated with significantly lower rates of major complications, including rebleeding, infarction, abscess formation, and contrast-induced nephropathy occurring in 10.7% of patients compared with 30.7% following distal embolization [24]. In this study, splenic infarction occurred in 33.3% of the coil group and 22.7% of the LOBO group. However, splenic infarction may reflect both the embolization approach and the underlying traumatic injury itself. Notably, the rates observed in our study were higher than those reported in more recent literature across different embolics [17].

There are key limitations associated with the study, including the retrospective design and the relatively small cohort size, which limit generalizability and the control of potential confounding that may exist between the two groups including injury severity score, non-random device allocation, and coil heterogeneity may therefore be clinically consequential. The study is also limited by heterogeneity in indications for splenic artery embolization and a lack of predefined criteria for splenectomy, which may introduce selection bias. Due to the small sample size, stratified analyses by indication were not performed, which may limit assessment of indication-specific outcome differences if present. Moreover, the cohort predominantly comprised patients undergoing SAE for traumatic splenic injury; therefore, the generalizability of these findings for non-traumatic SAE indications may be limited. Although the procedures were performed by experienced interventional radiologists, operator preference, and technique variability could have contributed to the increased fluoroscopy times observed in the coil group. Furthermore, while splenic infarction was observed less frequently in the LOBO group, this finding should be interpreted cautiously, given the limited follow-up imaging and the possibility that infarction may also occur as a direct consequence of the underlying traumatic injury itself, independent of embolization technique. Finally, real-world factors such availability, operator learning curve, as well as device cost, which were not assessed in this study, should be considered when selecting an embolic platform for distal splenic occlusion. Procedural costs, in particular, are challenging to compare reliably across patients because they are influenced by payer-specific factors such as insurance coverage, reimbursement structures, and negotiated payment rates [25].

## Conclusion

This preliminary retrospective analysis suggests that the LOBO device can be considered a safe and feasible embolic option for proximal splenic artery embolization, with procedural efficiency and short-term outcomes comparable to conventional embolics. Larger, prospective multicenter studies are warranted to confirm long-term efficacy and cost-effectiveness as compared to other devices used in proximal splenic artery embolization.

## Abbreviations

SAE: Splenic artery embolization
LOBO: Low-profile braided occlude
CT: Computed tomography
FDA: Food and drug administration
CIRSE: Cardiovascular and Interventional radiological society of Europe
